# Students’ attitudes toward and knowledge about snakes in the semiarid region of Northeastern Brazil

**DOI:** 10.1186/1746-4269-10-30

**Published:** 2014-03-27

**Authors:** Rômulo RN Alves, Vanessa N Silva, Dilma MBM Trovão, José V Oliveira, José S Mourão, Thelma LP Dias, Ângelo GC Alves, Reinaldo FP Lucena, Raynner RD Barboza, Paulo FGP Montenegro, Washington LS Vieira, Wedson MS Souto

**Affiliations:** 1 Departamento de Biologia, Universidade Estadual da Paraíba, Av. das Baraúnas, 351/Campus Universitário, Bodocongó, 58109-753 Campina Grande-PB, Brasil; 2 Departamento de Biologia, Universidade Federal Rural de Pernambuco. Rua Manoel de Medeiros, S/N, Dois Irmãos, 52171-900 Recife, Pernambuco, Brasil; 3 Departamento de Fitotecnia e Ciências Ambientais. Setor de Ecologia e Biodiversidade, Universidade Federal da Paraíba. Centro de Ciências Agrárias. Laboratório de Etnoecologia, Areia, Paraíba 58.397-000, Brasil; 4 Programa de Pós-Graduação em Ciências Biológicas (Zoologia), Departamento de Sistemática e Ecologia da Universidade Federal da Paraíba, 58051-900 João Pessoa, PB, Brazil

**Keywords:** Ethnoherpetology, Ethnozoology, Conservation, Snakes

## Abstract

**Background:**

Humans in various cultures have feared snakes, provoking an aversion and persecution that hinders conservation efforts for these reptiles. Such fact suggests that conservation strategies for snakes should consider the interactions and perceptions of the local population towards these animals. The aim of this study was to investigate students' perception of snakes and if attitudes and knowledge may differ according to gender and local residence (urban or rural).

**Methods:**

Data was collected in the second half of 2012 and consisted of questionnaires applied to 108 students in the Basic Education School in the municipality of Sumé, located in the semiarid region of Northeastern Brazil.

**Results:**

The male respondents recognized more species than female did. Part of the students affirmed to have a fear of snakes, especially women. Nearly half of respondents (49%) showed negative behaviour towards these animals, reflecting the influence of potential risk and myths associated with snakes, and supported by a limited knowledge about these animals and their ecological and utilitarian role. We find that the rural students recognized significantly more species than the urban students.

**Conclusions:**

Our results point to the need for educational interventions in order to increase knowledge about the positive aspects associated with snakes, seeking to minimize the influence of myths and beliefs that contribute to a strong aversion to snakes by the locals. Conservation strategies should therefore engage students but also teachers, who are key individuals in the process.

## Introduction

Because of overexploitation by humans combined with loss of suitable habitat, conflicts with people, invasions by exotic species and disease and other causes, many species of animals are now considered to be endangered in the wild [1]. Practically all threats which affect the animal species included in the IUCN Red List [2] are direct or indirectly associated to anthropogenic activities. This scenario represents a challenge in the quest for ways to exploit animal resources while at the same time minimizing the impact on animal species [1] and is evident that conservationists must understand not only the ecological, but also the cultural and economic interactions that link ecological and social systems into a common regional system, as well as understand the feedback that govern these interactions [3-6].

Conservation of biodiversity not only requires effective measures, such as the establishment of protected areas, legal regulations for the use of natural resources, and the control of introduced species [7], but also requires the dissemination of public information and education about native organisms, their value and the consequences of human activities on local biodiversity [1,7-9]. Achievements of conservation projects could improve if communication and biodiversity education are incorporated into the components of their design [10,11].

In the semiarid region of Brazil, the human population develops strong relationships with the local faunistic resources [12-18]. Many species interact with human communities and are hunted because of their utility (e.g., use as food, pets, and medicinal purposes) or because their conflicting relationships with the human population [12,16]. When we consider the animals involved in conflicts, snakes stand out [16]. The main reason for those conflicts relies on the fact that snakes eventually attack livestock and represent risks to human lives [13,16,19]. In addition, in this region, snakes inspire many myths, proverbs, and stories generated from the relationships with humans and passed down from generation to generation through oral traditions, thus, influencing how local people relate to these animals [16,20]. Moreover, these stories causing negative attitudes, thus these animals are associated with in fear and loathing by people in the community.

Because of this fear and negative perception, many people have very low interest in snakes and often perpetuate inaccurate myths [16,21]. This dislike is dangerous for both people and snakes because frightened people make irrational decisions that often result in snake death and/or an increased risk of a snakebite [16,22]. Snake persecution confounds conservation efforts. Even in some relatively undisturbed natural areas, snake numbers and diversity may be depressed because local people kill snakes [23]. In this context, it becomes evident that conservation strategies directed toward snakes should consider the interactions and perceptions of the local population towards these animals [16]. The ethnobiological approach is one way to investigate and establish relationships between the local and scientific knowledge in the school environment [24,25].

In view of this scenario, the present study is the first to investigate, from an ethnozoological perspective, the attitudes and knowledge of students about snakes in the semiarid region of Northeastern Brazil, aiming to provide baseline data for local conservation activities. The study focuses on the following questions: What is the students’ perception of snakes? Does this perception vary by their residence (urban or rural), gender and age of students? If students perceive snakes as dangerous and negative, how does this influence a conservation perspective? And what positive aspects of snakes are also recognized? Additionally, the conservation implications associated with perception of snakes are discussed, seeking to contribute to the conservation of the herpetofauna in the semiarid region of Northeastern Brazil.

## Methods

### Study area

**Figure 1:**
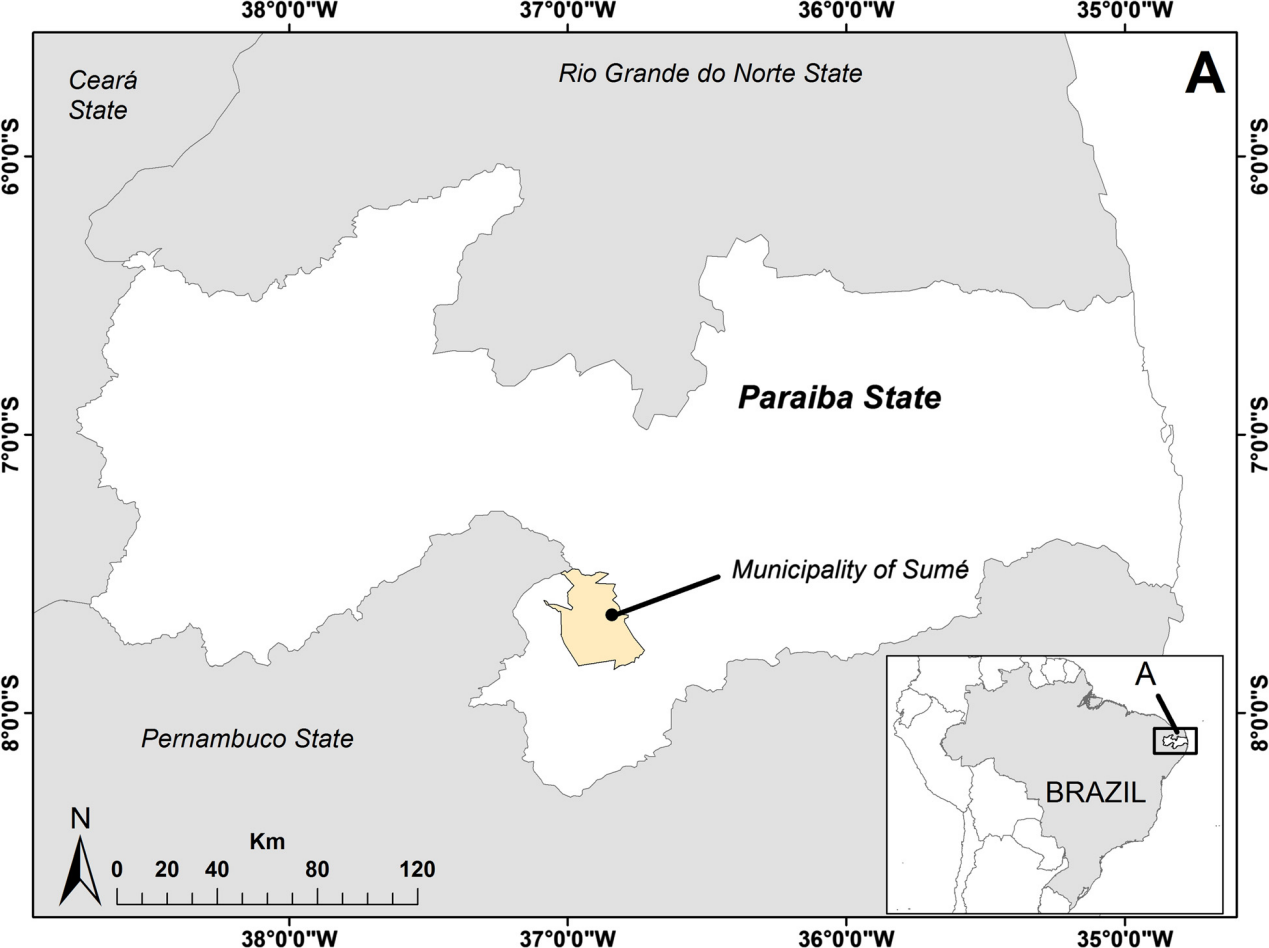
Map showing the studied area.

Field research was conducted in an educational institution located in the municipality of Sumé (07° 40′18 ″S and 36° 52′48″ W), Paraiba State, Northeastern Brazil (Figure 1). The municipality has an area of 867 Km^2^ and about 17,085 inhabitants. Information was gathered by the Agricultural School of Basic Education Department Evaldo Gonçalves de Queiroz, which assists students from 6th to 9th grade and residents of both rural (morning group) and urban area (afternoon group).

### Data collection

Data collection occurred in the second half of 2012. The information was obtained via a questionnaire to students of the Primary school (n = 108), aged between 11 and 1 years old. Among the respondents, 56 were female and 52 male. A total of 80 students reside in a rural and 28 in urban areas. Respondents’ level of education was as follows: 7th grade (n = 40), 8th grade (n = 40) and 9th grade (n = 28).

Before conducting the student interviews, visits were made, as well as, a formal permission request to the school in order to carry out the work. The objectives of the research were explained. Additionally, the consent form was presented and the school directors granted permission. Following this, visits were completed to the school classrooms and the consent form was given to the students to be taken to their parents so they could authorize the students’ participation in the study. Only after those procedures were complete, were data collected. The research was approved by the Ethics Committee of the State University of Paraiba (Protocol CAAE - 0026.0.133.000-10).

A questionnaire followed by visual stimuli [26] was presented to the students. This stimulus consisted of a board with pictures of different snake species that occur in the surveyed area. The inclusion of species arranged on the board was based on a zoological inventory carried out in Fazenda Almas, a conservation area of biodiversity considered a Private Natural Reserve of National Patrimony (PNRNP) [27], which is situated between the municipalities of Sumé (where the survey was conducted) and São José dos Cordeiros (Paraiba State, Brazil). The photos placed on the board were taken from a hunting herpetofauna catalogue of Caatinga semiarid, published by Alves et al. [16]. Besides the native species, images of three alien species and amphisbaenids (group of reptiles with similar morphology to snakes) were included as a control group.

The survey questionnaire was related to the recognition of the species, their possible importance or usefulness and whether the species was considered dangerous or not. Additionally, some questions about perception and attitudes towards snakes complemented the questionnaire (see Additional file 1).

### Data analysis

We used descriptive statistics for non-parametric data analysis. In order to verify the influence of gender and the location of respondents (urban or rural) in the number of recognized species, we used the Mann–Whitney U test [28]. To verify the influence of the interviewees’ age in relation to the number of recognized species, we used Kendall π correlation [29]; to assess whether the students’ education levels (7th, 8th and 9th grades of Primary school) influenced species recognition we used the Kruskal-Wallis H test [30]. The Mann–Whitney U test was also used to verify whether the respondents’ gender influences the perception of fear of snakes. For all tests, we used a significance level of 5% (p <0.05). Data were analyzed with SPSS© software version 20 [31].

## Results and discussion

The name “snakes” was the term used by all students interviewed to appoint all animals present on the board, including serpents (native or exotic) and amphisbaenians, revealing that serpentiform morphology is the main criterion used to recognize these animals, which are locally named cobra, even not belonging to the Ophidia suborder, a group that includes serpents in general. All snake species listed on the board (n = 27) were recognized by students; some of them were recognized by a larger number of students, especially *Micrurus ibiboboca* (recognized by 96 students, 88.9%), *Boa constrictor* (n = 75; 71.3%), *Crotalus durissus* (n = 65; 60.2%), *Philodryas olferssii* (n = 62; 57.4%) and *Oxyrhopus trigeminus* (n = 54; 50%). Even the exotic species were recognized by a significant number of students: *Naja siamensis* (n = 50; 46.3%), *Python molurus* (n = 35; 32.4%) and *Elaphe guttata* (n = 13; 12.0%). The recognition of the presented species is not surprising, since most species are native to the study region, allowing the possibility of contact or visualization of these snakes during everyday activities by the students themselves or by other people. Concerning exotic species, recognition may be related to the influence of the media (TV, internet), making it possible that these species can be viewed by students in movies, documentaries and internet sites, even though they do not occur in Brazil. This shows that the “local” knowledge or “traditional” knowledge may have a relationship to exogenous information, and even with scientific knowledge [32].

It is clearly noticed that the recognition of snake species is influenced by factors such as the size, color-pattern and poisonous potential, since the three snakes species known to a larger number of students were: *M. ibiboboca* (venomous snake with characteristic color-pattern), *B. constrictor* (the largest snake species that occurs in Northeast semiarid region) and *C. durissus* (one of the most venomous snakes that occurs in the region). Even when the students recognize the species pictured on the boards, discrepancies were found between the vernacular names given. For instance, eight respondents called the species *B. constrictor* rattlesnake “cascavel,” although this is a common name given to *C. durissus*. In a way, that divergence between vernacular names is not unexpected since the *folk* denominations of snakes may vary between different locations [16].

In general, female respondents recognize fewer species than male respondents (average of 5.7 and 8.3 species, respectively) (Table 1). Such difference is statistically significant (Mann–Whitney U test = 893, n (women) = 56, n (men) = 52, p <0.05)), and confirms previous studies which showed that the perception of animal varies with gender [33-35]. Farias and Alves [36], for instance, describe a case in which girls knew less about birds than boys do, and both group studied at a public school in the Metropolitan Region of Recife, Northeast Brazil. Kellert and Berry [33] point out that gender influences almost all dimensions of attitudes and knowledge about animals, and suggest that men and women have different emotional and cognitive orientations toward animals. Lindemann‒Matthies [37] showed that males generally like wild and exotic animals whilst females rather prefer pets. Kellert and Berry [33] found that women were more humane about animals than men, but they were also more negative in attitudes toward some animals than were men. Similar sex differences were also documented relating to humans’ fears of large carnivores; with females expressing a greater fear to phobic animals than males [38].

**Table 1:** Number of students who recognized the species presented on boards

**Species present on board images**	**Students (n /%)**	**Male (n)**	**Female (n)**
Native species			
*Micrurus ibiboca -* (Merrem 1820)	96 (89%)	47	49
*Boa constrictor* - (Linnaeus 1758)	75 (69%)	42	33
*Crotalus durissus* - (Linaeus 1758)	65 (60%)	38	27
*Philodryas olfersii* –(Lichtenstein 1823)	62 (57%)	35	27
*Oxyrhopus trigemenus* -(Duméril, Bibron & Duméril 1854)	54 (50%)	30	24
*Boiruna sertaneja* - (Zaher 1996)	41 (38%)	24	17
*Leptophis ahaetulla*- (Linnaeus 1758)	40 (37%)	22	18
*Oxybelis aeneus*- (Wagler 1824)	39 (36%)	27	12
*Liophis viridis*- (Gunther 1862)	32 (30%)	16	16
*Corallus hortulanus*- (Linnaenus 1754)	25 (23%)	16	9
*Bothropoides erythomela-* (Amaral 1923)	23 (21%)	11	12
*Leptodeira annulata*- (Linnaeus 1758)	18 (17%)	11	7
*Liophis poecilogyrus*- (Wied 1825)	16 (15%)	8	8
*Epictia borapeliotes*- (Vanzolini 1996)	16 (15%)	10	6
*Pseudoboa nigra*- (Duméril, Bibron & Duméril 1854)	11 (10%)	6	5
*Thamndynas teshypoconia*- (Cope 1860)	11 (10%)	6	5
*Apostepis cearensis*- (Gomes 1815)	10 (9%)	7	2
*Epicrates assisi*- (Machado 1945)	9 (8%)	5	4
*Philodryas nattereri*- (Steindachner 1870)	7 (6%)	3	4
*Pseudoboa nigra*- (Duméril, Bibron & Duméril 1854)	3 (3%)	2	1
*Xenodon merremii*- (Wagler 1824)	2 (2%)	2	0
*Thamnodynastes sertanejo*- (Bailey, Thomas & Silva-Jr 2005)	2 (2%)	2	0
***Exotic species***			
*Naja siamensis* Laurenti 1768	50 (46%)	30	20
*Python molurus* (Linnaeus 1758)	35 (32%)	20	15
*Elaphe guttata* Conant & Collins 1991	13 (12%)	8	5
***Amphisbaenas***			
*Amphisbaena alba*- (Linnaeus 1758)	65 (60%)	40	25
*Amphisbaena cf lumbricalis*- (Vanzolini 1996)	17 (16%)	12	5
*Amphisbaena vermicularis*- (Wagler 1824)	11 (10%)	8	3

In the surveyed area, the highest perception of snakes by boys may be related to the fact that the majority of respondents live in rural areas, where in general, the dominant role of men is with pastoral activities and subsistence agriculture, where it is common to encounter snakes, while women take care of household chores, where the possibility of encountering these animals is minimal. In fact, comparing the total snakes recognized by students of urban and rural areas, we find that the rural students recognized significantly more species than the urban students (Mann–Whitney U test = 797, n (rural) = 80, n (urban) = 28, p = 0.02).

Moreover, when we consider the age group and number of recognized snake species, we can see a slightly positive correlation (Kendall τ = 0.17, p <0.05), showing that age of the respondents did not strongly influence animal recognition. These results differ from previous studies that showed that age influences the perception of animals [33,39,40]. It should be emphasized, however, that in the present study, the results may be related to the slight variation in the respondents’ age in our sample (11–19 years, with average of 13.6), which makes it difficult to discuss this parameter accurately. The same situation was verified when considering the level of education, which did not affect the recognition of the number of snake species (Kruskal-Wallis H_(2)_ = 0.46; p > 0.05).

Most students (72.3%) distinguish between venomous and non-venomous species, an acknowledgment that may reflect information obtained from school. This situation differs from what has been recorded in ethnozoological researches in many localities of Northeast semiarid [16] and Brazil [19,41,42], where there is a widespread perception that most snakes species are considered poisonous, regardless of whether or not they have this characteristic. This is the same perception of a considerable part of interviewed students (n = 31, 28.7%), who affirmed not distinguish between venomous and non-venomous species. Among the 21 snake species that occur in the surveyed area and were presented to the students, only three are poisonous and most dangerous: rattlesnake “cascavel” (*Crotalus durissus*), Caatinga Lancehead “jararaca” (*Bothropoides erythromelas*) and real coral snake (*Micrurus ibiboboca*). Although most of the students recognize that most species are not poisonous, they are considered aggressive and dangerous, a fact that presents a serious conservation problem as it stimulates the indiscriminate serpents kill, regardless of whether or not they are poisonous. It should be highlighted that only a marginal number of local species are being pointed out as responsible for human deaths in Northeastern Brazil [43], in the surveyed area, as well as in other areas of Northeast semiarid [16] and Brazil [19,41,42].

The aversion to snakes also extends to the amphisbaenians, which are popularly known as serpents due to their serpentiform morphology. Respondents recognize and classify these animals as snakes, naming them mostly as “two headed snakes.” This kind of perception contributes to the development of a negative behaviour regarding these animals. A similar situation was reported by Pinto et al. [44], who completed ethnozoological research in the Minas Gerais State and found that, besides snakes, amphisbaenians and lizards of the genus *Ophiodes* and *Heterodactylus* are also arbitrarily killed by locals due to classify them as serpents. This shows a strong connection between knowledge and behaviour, an important aspect in ethnoecological studies in general [45].

Most respondents (n = 66; 61.1%) affirmed that they were fearful of snakes. Such aversion is more frequent among women (n = 49; 74%), while 32.7% (n = 17) of men also have this perception (Mann–Whitney U = 684, n (_men_) = 52, n (_women_) = 56, p <0.05; ΣR_women_ = 3824, ΣR_men_ = 2062), showing a trend reported in other studies which pointed out that gender differentiates knowledge of and attitudes toward animals [7,33,46]. Similar to our results, Prokop et al. [47], in research on students’ attitudes regarding snakes, verified that fear of snakes is higher in women than in men. Likewise, this situation was also reported in relation to other animals such as spiders [48] and bats [48,49]. It is noteworthy that regardless of gender, there is a great aversion to snakes, which is not surprising as the people’s negative perception in relation to these animals is common in the Northeast [16].

Snakes have been feared by humans of many cultures, particularly due the fact that many snakes are deadly venomous [50]. In the surveyed area, the lethal potential of the serpents certainly contributes to the spread of the fear of these animals [16]. However, most species occurring in the semiarid region are not poisonous, meaning that many snake accidents may not result in the victims’ death. This reality, though, is ignored by much of the population who cannot distinguish between venomous and non-venomous species, and thus, consider all these animals as dangerous and harmful [16]. This same approach was observed among part of the interviewed students who demonstrate uncertainty on the differentiation between species that are venomous and those that are not, although the majority of respondents (n = 77) agreed that not all snakes are poisonous.

Importantly, the fear of snakes is not only related with real risks that some species represent, but also to the legends, beliefs, fables, myths and other cultural aspects linked to these animals [1,16,19,51,52]. Among the most relevant cultural features, religious beliefs and practices have long influenced the interaction between serpents and people [53,54], and in the Northeast semiarid region it is no different [16,52]. In this region, Catholicism is among the most widespread religions, and as well as other expressions of Christian faith, it has influenced the attitudes towards the local wildlife [55,56]. In this scenario, biblical passages such as in the book of Genesis, in which a serpent deceives Eve, the first woman created by God, so that she eats the fruit of the forbidden tree in order to have the discernment of good and evil, can contribute to the bad reputation of snakes and is one of the reasons there is such an aversion to these animals in the region [52]. These negative perceptions of snakes are widespread in largely ophiophobic Judeo-Christian societies, which condemn snakes for their perceived treachery against humans as represented by Adam and Eve in biblical times [57-59]. In addition, there are several other myths and tales not related to religion that contribute to the persecution of snakes in the Brazilian semiarid region [16,52]. It is important to stress, however, that not every animal culturally associated with evil is hunted indiscriminately, as pointed by Marques [55] and Farias et al. [56].

Assuming eventual encounters with snakes, almost half of the students (n = 53, 49%) responded in such a way that indicates that they would kill the animal; 22 of affirmed they would do it by themselves and 31 stated they would call someone to accomplish this task. Among others respondents, 48 would choose to let the snake live and 5 would make the snake go back to the bushes. It is noticeable that the part of respondents have negative attitudes towards these animals, which reflects common practices in the Northeast semiarid region where snakes are frequently killed during daily activities of local people, regardless of species [16]. Two respondents did not answer this question. It is interesting that there was a development of negative attitudes (as is common in the Brazilian semiarid region), but also positive, which may be a result of the close contact with scientific information disseminated in books, television programs and internet sites [32].

Positive aspects related to snakes were mentioned by 32 students, of which attributed some importance or utility of any value to these animals (Table 2). Thirteen students cited at least two main aspects of importance of these animals: a) some species are important once they feed on other snakes and other poisonous animals, and b) the poison works as a “medicine.” The first statement, despite being identified as a positive aspect, reveals the antipathy that local people have on all kind of snakes. The second statement, which imparts medicinal value to snakes, is supported by the medical literature that recognizes that reptiles have been used as sources of drugs for modern medical practices [60,61]. Reptiles’ venoms are complex mixtures of bioactive molecules [62]. Moreover, the venom of snakes belonging to the families *Viperidae* and *Elapidae* contain analgesic substances that are stronger than morphine and have been used to treat terminal cancer patients [63].

**Table 2:** Examples of ecological importance utility value of snakes according to respondents

**Importance**	**No. of citations**
Important for nature	8
It is part of the food chain, controlling the populations of other animals	5
Prevent the growth of pests such as rats	2
Eat other snakes and other poisonous animals	13
**Utility value of snakes**	
“The poison serves to make medicine”	13
Used as food	2
“The rattle can be used for some stuff”	2
Used in folk medicine	3
As pet	1
**Total**	**21**

### Final considerations and implications for conservation

The present study provides evidence that snakes are perceived by many of the students as being harmful and dangerous, and most students face a major aversion to snakes. These perceptions encourage negative attitudes, especially related to the attempt to kill whenever a snake is found. These negative attitudes reflect the influences of a potential threat and some myths associated with snakes. It is also due to limited knowledge about these animals and their ecological and utilitarian role. Similar situations occurs not only in Brazil but is spread in different locations worldwide, making snakes among the most disliked animals; they trigger very strong levels of fear and destructive behaviours [47,64-67].

On the other hand, a significant portion of students admitted positive aspects about snakes, highlighting their ecological and economical role. This positive perception is important from a conservationist perspective and should be encouraged, seeking to minimize the influence of myths and beliefs that contribute to a strong hatred for these animals. Conservation strategies should therefore engage students but also teachers, who are key individuals in the process.

Clearly, negative perceptions make the snakes among the most difficult animals to conserve. The conservation of snakes is more difficult than for other vertebrate groups because of the generally bad reputation that snakes have in many regions of the world [53]. As pointed out by Bevins and Bitgood [68], a large number of individuals fear snakes, believe that snakes are dangerous, and are not aware of the important role of snakes in the ecosystem. That same trend occurs in the region where this study was conducted, the results of which indicate the urgent need for environmental education strategies, thus, reinforcing the positive aspects related to snakes, which as can be seen, is present among the students interviewed. According to Kellert [69], education plays a crucial role in informing people about organisms and the environment, helping to develop responsible attitudes and behaviours. As the aversion on snakes is quite common throughout Brazil, it is important to note that educational programs should be implemented not only in schools but also through campaigns in museums and zoos, thus aiming to reach the general public. Specifically in the semiarid region of Brazil, where this research was conducted, one challenge facing snake conservation is changing the way people perceive snakes, highlighting their ecological importance and clarifying myths about the potential risk they represent.

## Competing interests

The authors declare that they have no competing interests.

## Authors’ contributions

RRNA, VNS, DMBMT, JVO, JSM, TLPD, AGCA, RFPL, RRDB, PFGPM, WLSV and WMSS–Writing of the manuscript, literature survey and interpretation, and analysis of taxonomic aspects; VNS, RRNA–Ethnozoological data. All authors read and approved the final manuscript.

## Supplementary Material

Additional file 1 Questionnaire used for data collection. Click here for file
