# Supplementary material for: Students’ attitudes toward and knowledge about snakes in the semiarid region of Northeastern Brazil
Source: J Ethnobiol Ethnomed. 2014 Mar 27;10:30. doi: 10.1186/1746-4269-10-30 (PMC3986856; doi:10.1186/1746-4269-10-30)
Supplement: Additional file 1 — Questionnaire used for data collection. [file 1746-4269-10-30-S1.docx]

**Additional file 1**

**QUESTIONNAIRE**

**School: ______________________________________________________**

**Age: _________ Gender: M ( ) F ( ) Grade: ___________________**

**Dwelling-place: urban area ( ) rural area ( )**

**1. What is a snake?**

_______________________________________________________________

_______________________________________________________________

**2. Are you afraid of snakes?**

( ) yes ( ) no

If so, why?

_______________________________________________________________

_______________________________________________________________

**3. Do you consider all snakes as venomous?**

( ) yes ( ) no

If not, how do you differentiate a poisonous snake from a non-poisonous one?

_______________________________________________________________

_______________________________________________________________

**4. Which of the following actions you would take in an eventual encounter with a snake:**

a) ( ) let the snake go away.

b) ( ) scare off the snake to the bush

c) ( ) tries to capture it

d) ( ) asks someone to kill it

e) ( ) you kill the snake.

**5. Have you ever suffered a snakebite?**

( ) yes ( ) no

If so, what was the injured body part?

______________________________________________________________

Do you know someone who has ever been bitten by snakes? What happened to that person?

______________________________________________________________

Thank you!
